# Operative and non‐operative management can result in acceptable long‐term outcomes for isolated posterior cruciate ligament injuries in paediatric patients

**DOI:** 10.1002/jeo2.70421

**Published:** 2025-09-11

**Authors:** Morgan G. Batley, Nathan Chaclas, Katherine Ashe, Caroline L. Kim, Theodore J. Ganley, Kathleen J. Maguire, Brendan A. Williams

**Affiliations:** ^1^ Department of Orthopaedics The Children's Hospital of Philadelphia Philadelphia Pennsylvania USA; ^2^ Perelman School of Medicine at the University of Pennsylvania Philadelphia Pennsylvania USA

**Keywords:** paediatric orthopaedic injury, paediatric posterior cruciate ligament injury, paediatric sports medicine, posterior cruciate ligament injury, posterior cruciate ligament treatment

## Abstract

**Purpose:**

The purpose of this study was to evaluate the injury characteristics, treatment, and outcome of paediatric patients with isolated posterior cruciate ligament (PCL) injuries treated at a single center. Authors hypothesised that both treatment cohorts would successfully return to sport participation with a low risk of PCL retear or ongoing knee‐related symptoms.

**Methods:**

A retrospective review and cross‐sectional outcomes assessment were performed identifying patients <18 years old with PCL injuries from a single treatment center between 2015 to 2021 using ICD‐10 coding. Patients with concomitant collateral or cruciate ligament injury were excluded. Studied variables included patient demographics, injury characteristics, treatment strategies, and patient outcomes (PROMIS, IKDC and Lysholm scores and reinjury). A cross‐sectional follow‐up survey was distributed to all patients in spring 2023. Descriptive statistics were performed for continuous and categorical outcomes. Bivariate analyses were performed on all variables between operative and nonoperative treatment groups. A non‐response analysis was completed to evaluate non‐response bias of the cross‐sectional cohort due to the incomplete response rate.

**Results:**

Twenty‐four patients meeting inclusion criteria were identified. Injured patients had a mean age of 13.2 years old and were predominantly male (67%). Injuries most occurred during sport participation (75%) and were managed nonoperatively (67%). Overall, there were no differences between treatment groups regarding complications or return to sport. Eleven (46%) completed the cross‐sectional outcomes assessment at an average of 4.7 years from injury. Most patients had successfully returned to sport without sustaining an ipsilateral knee injury with patient reported outcome scores within normative ranges.

**Conclusion:**

These results suggest that both operative and non‐operative treatment strategies are reasonable in the short‐ and long‐term management of paediatric PCL injuries.

**Level of Evidence:**

Level IV.

**Abbreviations**
BMI: body mass index
CPT: current procedural terminology
ICD10: International Statistical Classification of Diseases, 10^th^ revision
IKDC: International Knee Documentation Committee
OR: operating room
PCL: posterior cruciate ligament
PROMIS: Patient Reported Outcome Measurement Information Systems
REDCap: Research Electronic Data Capture

## INTRODUCTION

Posterior cruciate ligament (PCL) injuries are uncommon in comparison to other cruciate or collateral ligament injuries of the knee and rarely occur in isolation [8]. Both high‐energy trauma such as dashboard injuries in motor vehicle accidents and sport‐related injuries are the most common mechanisms of isolated PCL tears [7, 10, 18]. Existing evidence guiding the management of PCL tears in isolation is limited; however, general consensus supports initial conservative measures including bracing and activity restriction as well as gradual activity progression under the guidance of physical therapy [6, 13, 16]. Operative repair or reconstruction is favoured for non‐isolated tears, ongoing instability after a trial of conservative measures, and for multi‐ligamentous knee injuries [6, 8, 12].

Existing evidence regarding the management of isolated PCL injuries is largely based on adult cohorts given the epidemiologic patterns of this injury [10, 16, 18]. However, these injuries do occur in skeletally immature paediatric patients, with evidence that a decreased posterior tibial slope may increase odds of injury [7, 15]. Published reports of injury characteristics and treatment‐related outcomes for isolated PCL injuries in paediatric patients are sparse [5, 7, 12]. Prior reports suggest both operative and nonoperative management strategies can effectively guide patients back to sport participation with low risk of reinjury or prolonged knee dysfunction [3, 5, 7, 12].

In order to bolster the existing evidence base regarding paediatric PCL injuries, the goal of the present study was to retrospectively review and cross‐sectionally evaluate patients treated for this pathology at the institution of study. Study aims were (1) To evaluate the injury characteristics, treatments and long‐term outcomes of isolated PCL injuries and (2) To compare these characteristics and outcomes between patients treated operatively and nonoperatively. Based on prior literature, study authors hypothesised that both treatment cohorts would successfully return to sport participation with a low risk of PCL retear or ongoing knee‐related symptoms.

## METHODS

A retrospective review of paediatric patients (<18 years old at the time of injury) treated for isolated PCL injuries (defined as PCL injury without other collateral or cruciate injury) was performed after approval from the center's institutional review board for all aspects of this study. Chart review identified patients treated at a single urban tertiary care center and outpatient clinic between 2015 and 2021. A billing query was utilised to identify all patients with associated current procedural terminology (CPT) and International Statistical Classification of Diseases, 10th revision (ICD10) codes, detailed in Supporting Information: Appendix 1. Manual chart review of the resultant patient list was conducted to evaluate for PCL injury. Patients with concomitant cruciate or collateral ligamentous injury, as well as those with underlying musculoskeletal abnormalities, were excluded.

Patient demographic and anthropometric data including age, sex, height, weight, Body Mass Index (BMI), and race/ethnicity were recorded. Injury characteristics including mechanism, laterality, location, and composition were gathered from the electronic medical record. Tear thickness (partial versus full) was defined according to the musculoskeletal radiologists review in the advanced imaging report. Time delay to treatment was recorded, and radiographic imaging was reviewed to capture regional physeal status (open/closing/closed). Temporal rehabilitative milestones included weeks until full weight bearing, range of motion, isolated hamstring strengthening, running/impact activities, and full return to sport permitted. Patient decision to return to sport (full return to play) was deduced from physician and physical therapist determination of readiness for sport. Functional outcomes, including weeks of non‐functional bracing, functional testing metrics, and presence of functional bracing on return to sport were recorded. Clinical outcomes including knee range of motion, Lachman and posterior drawer stability were recorded. Within the operative subcohort, time to surgery, operative approach, construct, fixation and any concomitant procedures were recorded. Treatment complications included continued instability defined by persistent high‐grade posterior drawer laxity appreciated by the provider on physical exam or subjective knee laxity perceived by the patient, re‐tear/reinjury, and return to the operating room (OR) for the operative subcohort.

Ethical approval for retrospective and cross‐sectional arms of this investigation was obtained for our Institutional Review Board prior to initiation of the study. All patients were contacted with the intent of collecting several follow‐up cross‐sectional survey instruments, including Patient Reported Outcome Measurement Information System (PROMIS) t‐scores, International Knee Documentation Committee (IKDC) scores, and Lysholm scores. All questionnaires have been previously validated and utilised in the paediatric and adult orthopaedic sports literature [1, 8, 11, 13]. A patient questionnaire regarding subsequent injury and activity level was also included. Surveys were administered through Research Electronic Data Capture (REDCap) online survey software and completed by patients. Age at the time of survey completion and time from treatment was collected for analysis.

### Statistics

The study timeline was set to include all reliable electronic medical record information, and all eligible patients were included over this timeframe. Given this, the utility of an a‐priori sample size calculation was rendered low. Descriptive statistics were performed for continuous and categorical outcomes. Bivariate testing for significance included two‐sample independent‐test and Wilcoxon rank sum test, Pearson *χ* ^2^ test, and Fisher exact test. A non‐response analysis was completed to evaluate non‐response bias of the cross‐sectional cohort due to the incomplete response rate. Please refer to Supporting Information: Appendix 2 for a completed STROBE Checklist for cross‐sectional studies [2].

## RESULTS

**Figure 1:**
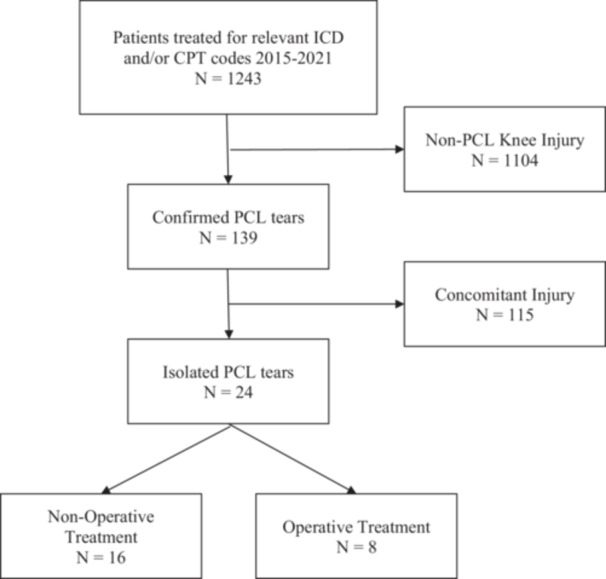
Study participant selection process. CPT, current procedural terminology; ICD, International Statistical Classification of Diseases; PCL, posterior cruciate ligament.

**Figure 2:**
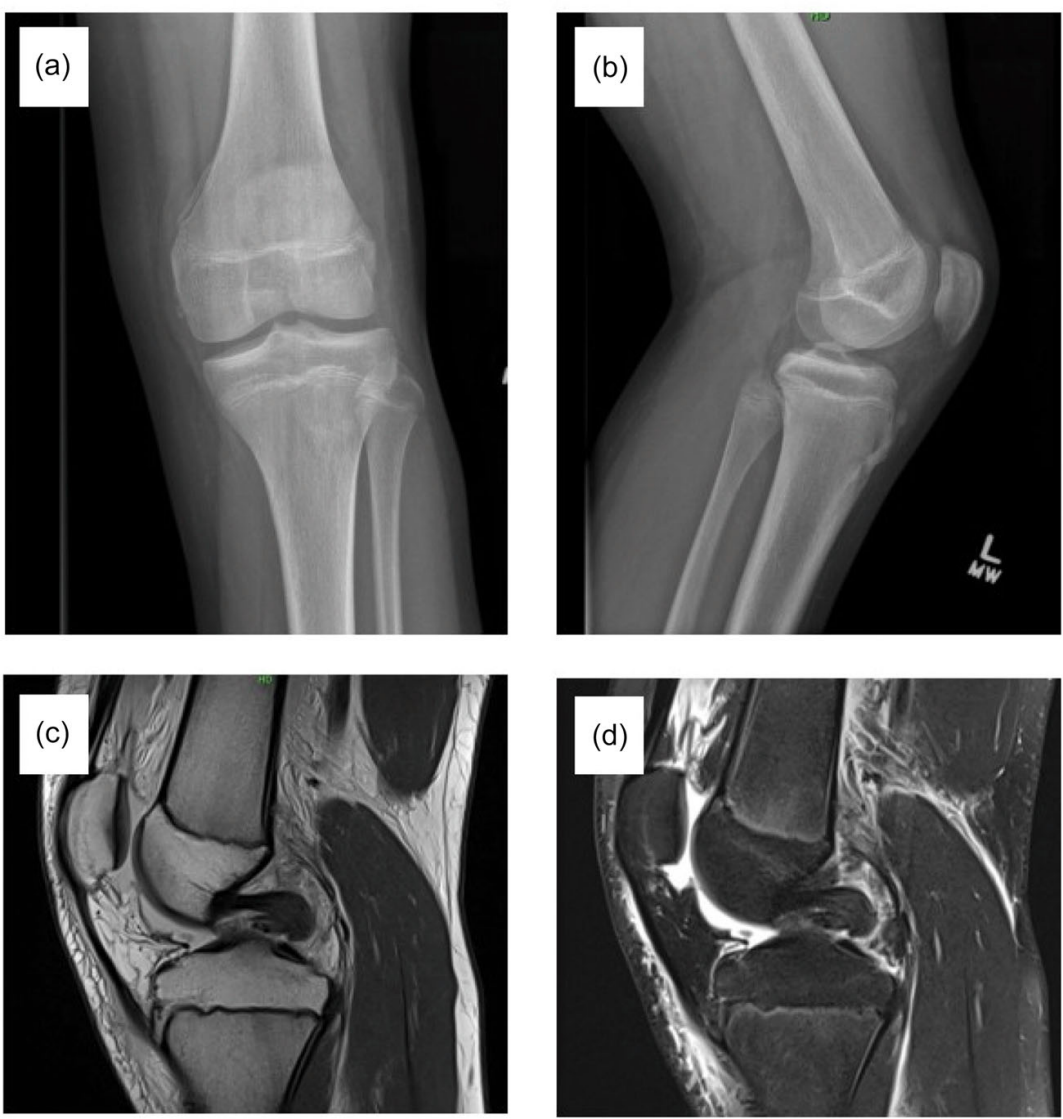
Radiographic imaging of a 14‐year‐old male who sustained a complete PCL tear while water tubing. The patient successfully returned to sports after 7 months of physical therapy and sustained no recurrent injuries at latest follow‐up. (a) Anterior/posterior view x‐ray. (b) Lateral view x‐ray. (c) Sagittal view T1 weighted MRI. (d) Sagittal view T2 weighted MRI. MRI, magnetic resonance imaging; PCL, posterior cruciate ligament.

**Figure 3:**
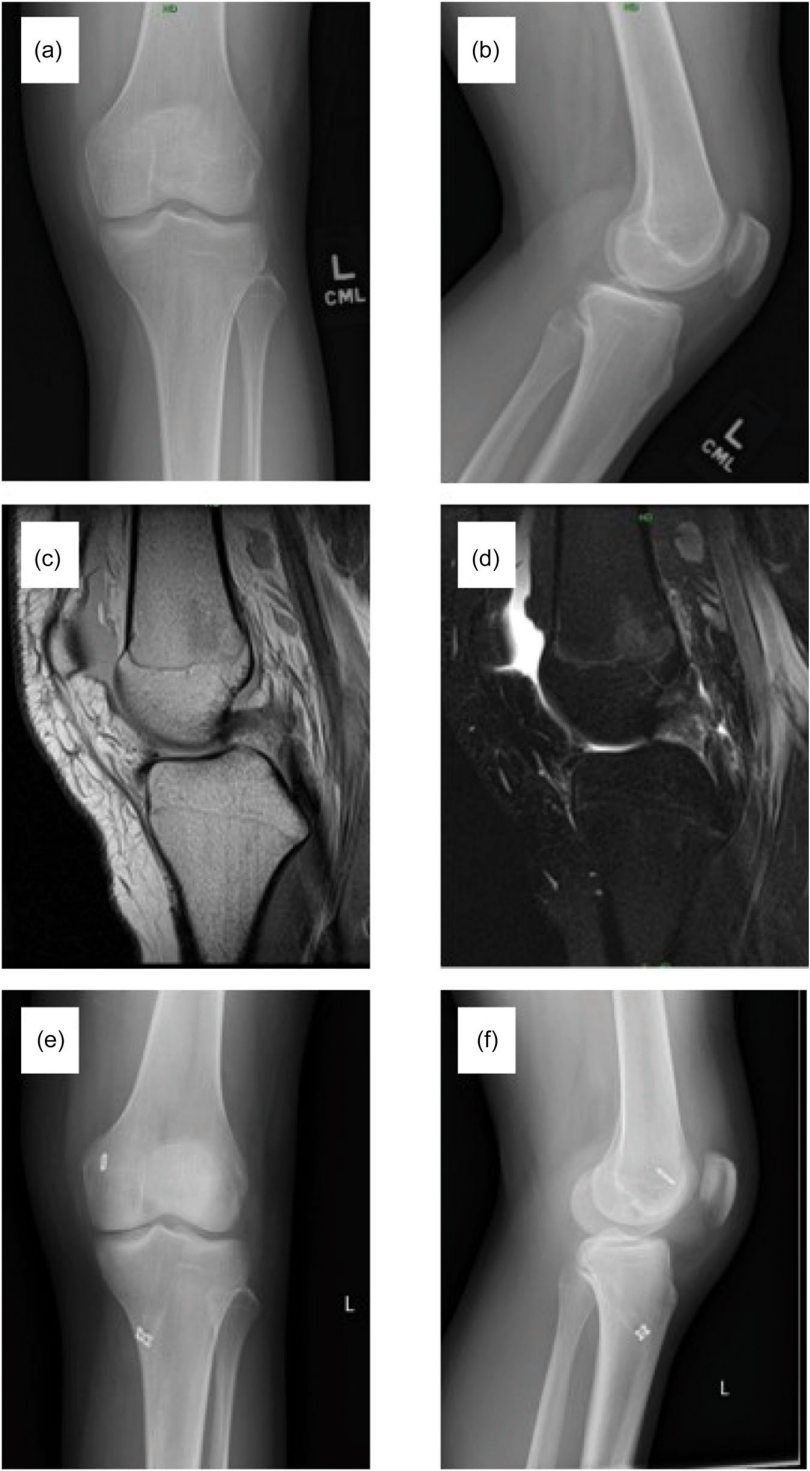
Radiographic imaging of a 17‐year‐old male who sustained a complete PCL tear while skateboarding that was managed surgically with PCL reconstruction using tripled posterior tibial tendon allograft. The patient successfully returned to sports after 9 months of physical therapy and sustained no recurrent injuries at latest follow‐up. (a) Pre‐operative anterior/posterior view x‐ray. (b) Pre‐operative lateral view x‐ray. (c) Sagittal view T1 weighted MRI. (d) Sagittal view T2 weighted MRI. (e) Post‐operative anterior/posterior view x‐ray. (f) Post‐operative lateral view x‐ray. MRI, magnetic resonance imaging; PCL, posterior cruciate ligament.

Study criteria identified 24 patients with isolated PCL injury during the study period (Figure 1). Patients were predominantly white (75%) males (67%) with a mean age of 13.2 ± 3.4 years (Table 1). Injuries most commonly occurred during sports participation (75%) with a contact mechanism (67%). Patients were evenly distributed across physeal maturity groups (Table 2). The most common injuries were tears (88%) to the middle third of the ligament (46%). Most patients were managed non‐operatively (67%); however, those with full thickness tears more commonly underwent operative intervention (Table 2). All patients underwent physical therapy; however, protocols demonstrated notable variation (Table 3). Radiographic imaging demonstrating nonoperatively and operatively managed full thickness PCL tears can be found in Figures 2 and 3.

**Table 1:** Demographic and anthropometric characteristics.

Variable	Total	Non‐Operative	Operative	*p*‐Value
	(*N* = 24)	(*N* = 16)	(*N* = 8)	
Age (years)	13.2 ± 3.4	13.3 ± 3.2	12.9 ± 3.9	0.79
Range (years)	4–17	4–16	7–17	
Sex				
Male	16 (67)	10 (63)	6 (75)	0.67
Female	8 (33)	6 (37.5)	2 (25)	
Race				
Black	4 (17)	2 (13)	2 (25)	0.28
White	18 (75)	13 (81)	5 (63)	
Other	2 (8)	1 (6)	1 (12)	
Ethnicity				
Hispanic/Latinx	2 (8)	2 (13)	0 (0)	0.54
Non‐Hispanic/Latinx	22 (92)	14 (87)	8 (100)	
BMI (kg/m^2^)	23.5 ± 4.9	23.9 ± 5.3	22.9 ± 4.5	0.64

*Note*: Internal values are *N* (%) or mean ± standard deviation.

Abbreviation: BMI, body mass index.

**Table 2:** Skeletal maturity and injury characteristics.

Variable	Total	Non‐Operative	Operative	*p*‐Value
	(*N* = 24)	(*N* = 16)	(*N* = 8)	
Activity during injury				
Sport	18 (75)	13 (81)	5 (63)	0.36
Non‐Sport	6 (25)	3 (19)	3 (37)	
Mechanism				
Contact	16 (67)	10 (63)	6 (75)	0.57
Non‐contact	8 (33)	6 (38)	2 (25)	
Physeal Status				
Open	12 (50)	9 (56)	3 (37)	0.66
Closing	10 (42)	6 (38)	4 (50)	
Closed	2 (8)	1 (6)	1 (12)	
Laterality				
Right knee	11 (46)	8 (50)	3 (37)	0.68
Left knee	13 (54)	8 (50)	5 (63)	
Location of tear				
Proximal	7 (29)	3 (19)	4 (40)	0.26
Mid	11 (46)	8 (50)	3 (37)	
Distal	6 (25)	5 (31)	1 (12)	
Tear type				
Ligamentous	21 (88)	15 (94)	6 (75)	0.25
Avulsion	3 (12)	1 (6)	2 (25)	
Tear thickness				
Partial tear	13 (54)	12 (75)	1 (12)	**<0.01**
Full	11 (46)	4 (25)	7 (88)	
Concurrent injury^a^				
Yes	12 (50)	7 (44)	5(63)	0.67
No	12 (50)	9 (56)	3 (37)	

*Note*: Internal values are *N* (%) or mean ± standard deviation.

a Concurrent injuries included meniscus tear and/or meniscus root injury, tibial fracture and/or bony contusion, gastrocnemius strain, and medial femoral condyle contusion.

**Table 3:** Rehabilitation and treatment‐related complications during clinical follow‐up.

Variable	Total	Non‐operative	Operative	*p*‐Value
	(*N* = 24)	(*N* = 16)	(*N* = 8)	
Functional bracing prescribed	19 (79)	12 (75)	7 (88)	0.63
Weeks to full weight bearing	2.0 ± 2.4	1.0 ± 1.9	3.9 ± 2.0	**<0.01**
Weeks to full range of motion	2.2 ± 2.5	1.6 ± 2.5	3.4 ± 2.1	0.08
Weeks to isolated hamstring strengthening	5.4 ± 4.8	3.3 ± 13.4	9.4 ± 5.3	**0.01**
Months to impact activities	3.5 ± 1.4	3.1 ± 1.1	4.3 ± 1.5	0.08
Months to return to sport	7.1 ± 4.1	6.1 ± 2.7	8.9 ± 0.4	0.06
Months of clinical follow‐up	15.0 ± 17.8	8.8 ± 9.8	27.5 ± 23.8	0.07
Complications				
Continued instability	0 (0)	0 (0)	0 (0)	0.58
Re‐tear/re‐injury	0 (0)	0 (0)	0 (0)	
Return to OR (*N* = 8)	2 (25)	‐	2^a^ (25)	

*Note*: Internal values are *N* (%) or mean ± standard deviation.

Abbreviation: OR, operating room.

a Returns to OR due to stiffness (manipulation under anaesthesia) and displaced anchor, respectively.

Nonoperatively management patients were permitted to be full weight bearing significantly sooner than their operative counterparts (1 ± 1.9 versus 3.9 ± 2 weeks, *p* < 0.01) and were advanced to isolated hamstring strengthening earlier than operative patients (3.3 ± 13 for operative patients versus 9.4 ± 5.3 weeks for nonoperative patients, *p* = 0.01). No other significant differences in rehabilitation timeline were observed between groups. The majority of patients (79%) utilised a functional brace upon return to activities. Treatment and outcome characteristics are further detailed in Tables 3 and 4.

**Table 4:** Surgical technique and time to operative delay and approach.

Variable	Operative subcohort (*N* = 8)
Time from injury to surgery (months)	14.5 ± 28.5
Approach	
Arthroscopic	7 (88)
Open technique	1 (12)
Reconstructive	6 (75)
Repair graft choice	2 (25)
Autograft	5 (62)
Allograft	1 (12)
Autograft type	
Gracilis triple 6 strand	2 (40)
Semitendinosus and gracilis triple 6 strand	2 (40)
Quadriceps	1 (20)
Graft fixation method	
Cortical suspension	6 (100)
Concurrent procedures^a^	3 (38)

*Note*: Internal values are *N* (%) or mean ± standard deviation.

a Concurrent procedures included arthroscopy with chondroplasty of medial femoral condyle, meniscus root repair.

Within the operative cohort, an initial attempt at conservative management was highly variable as reflected by the lack of uniform delay to surgery. Notably, one patient completed a significant course of conservative management but ultimately underwent operative management for persistent symptoms. Intra‐operatively, arthroscopic management was most common (88%) with reconstruction (75%) being the preferred approach. Autograft was the most common graft choice (62%).

Two complications occurred within the operative cohort: one instance of stiffness requiring a return to the OR for manipulation under anaesthesia and one displaced anchor. Patients reliably progressed through full weight bearing, range of motion, hamstring strengthening, impact activities, and return to sport by 7.1 ± 4.1 months. Mean final clinical follow‐up was 15 ± 17.8 months (range: 1–72 months) (nonoperative cohort, 8.8 ± 9.4 months (range: 1–31 months); operative cohort, 27.5 ± 22.28 months (range: 6–72 months); *p* = 0.06).

**Figure 4:**
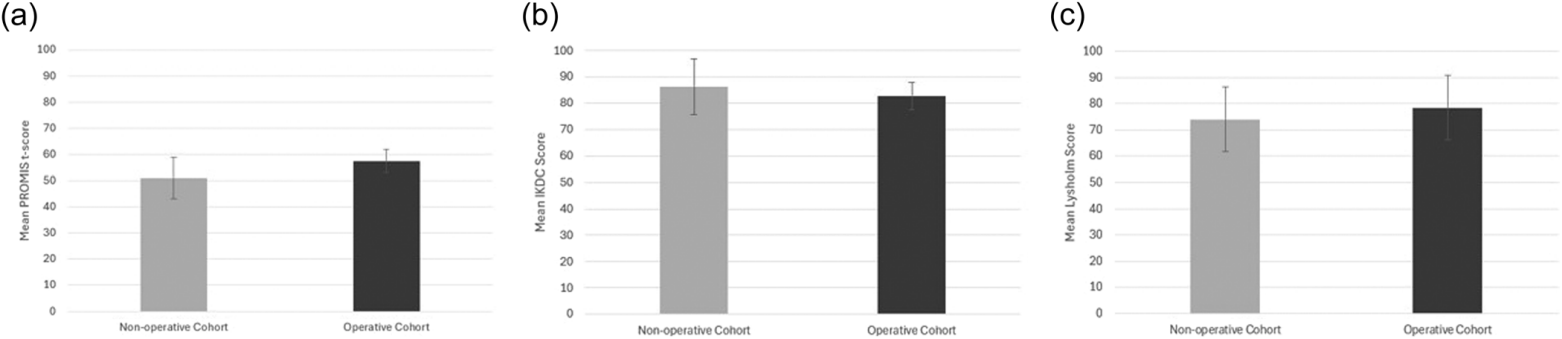
Mean patient reported outcome scores at mid‐term follow‐up. (a) Patient Reported Outcome Measurement Information Systems (PROMIS) t‐scores. (b) International Knee Documentation Committee scores. (c) Lysholm scores. Error bars represent standard deviation.

Cross sectional surveys were administered 4.7 ± 2.8 years after the initial injury, at a mean patient age of 17.3 ± 4.9 years. All patients were contacted. The response rate was 46%. Amongst responders (Table 5), a majority successfully returned to sport with no statistically significant difference between cohorts. No new complications were reported for either cohort. PROMIS mobility, IKDC, and Lysholm instrument scores did not differ significantly between the operative and non‐operative cohorts (Figure 4). Results of non‐responder analyses indicate low risk of non‐response bias (Table 6).

**Table 5:** Treatment outcomes at mid‐term follow‐up.

Variable	Total (*N* = 11)	Non‐operative (*N* = 6)	Operative (*N* = 5)	*p*‐Value
Age at survey response (years)	17.3 ± 4.9	15.8 ± 2.2	19.2 ± 6.7	0.33
Injury to survey response (years)	4.7 ± 2.8	3.3 ± 2.2	6.4 ± 2.8	0.08
Successful return to sport after injury	8 (73)	4 (67)	4 (80)	0.58
Subsequent ipsilateral knee injury	0 (0)	0 (0)	0 (0)	‐

*Note*: Internal values are *N* (%) or mean ± standard deviation.

**Table 6:** Nonresponder analysis of cross‐sectional outcomes assessment.

Variable	Responder (*N* = 11)	Non‐responder (*N* = 13)	*p*‐Value
Age (years)	12.6 ± 4.3	14.4 ± 1.9	0.22
Race			
Black	1 (9)	4 (31)	0.13
White	10 (91)	7 (54)	
Other	0 (0)	2 (25)	
Ethnicity			
Hispanic/Latinx	0 (0)	2 (15)	0.48
Non‐Hispanic/Latinx	11 (100)	11 (85)	
BMI	22.5 ± 5.1	24.4 ± 4.7	0.36
Mechanism of injury			
Sport	7 (64)	11 (85)	0.36
Non‐sport	4 (36)	2 (15)	
Contact	7 (64)	9 (69)	1.00
Non‐contact	4 (36)	4 (31)	
Physeal status			
Open	6 (55)	6 (46)	0.84
Closing	4 (36)	6 (46)	
Closed	1 (9)	1 (8)	
Laterality			
Right knee	2 (18)	9 (69)	**0.02**
Left knee	9 (82)	4 (31)	
Tear type			
Ligamentous	8 (73)	13 (100)	0.08
Avulsion	3 (27)	0 (0)	
Tear thickness			
Partial tear	4 (36)	9 (69)	0.22
Full	7 (64)	4 (31)	
Injury complex			
Isolated PCL	6 (55)	6 (46)	1.00
Concurrent injury	5 (45)	7 (54)	
Treatment			
Non‐operative	6 (55)	10 (77)	0.39
Operative	5 (45)	3 (23)	

*Note*: Internal values are *N* (%) or mean ± standard deviation.

Abbreviation: BMI, body mass index; PCL, posterior cruciate ligament.

## DISCUSSION

This study highlights the characteristics and outcomes of isolated PCL injuries in paediatric patients and serves to bolster the limited available evidence guiding clinical decision‐making on this topic. In support of the present study's initial hypothesis, both operative and nonoperative treatment groups achieved successful return to sport in a similar timeframe with low rates of complication. Within a limited sub‐cohort with long‐term follow‐up, there were no PCL re‐tears and patient‐reported and knee functional outcomes in both treatment groups were within normative range for this age‐group indicating good long‐term outcomes without treatment strategies.

Albeit rare, isolated PCL injuries do occur in the paediatric population, even amongst skeletally immature patients. In contrast to adult cohorts, the present study identified that injuries sustained from sport participation are more common than trauma‐related mechanisms. Nonoperative and operative treatment strategies resulted in successful return to sport in similar timeframes and with low rates of treatment failure or reinjury and good patient reported and knee functional outcome scores at midterm cross‐sectional follow‐up. These findings support the consideration of both treatment strategies for this rare injury type in a unique cohort of patients.

Considerable heterogeneity exists in management practices across the literature regarding the treatment of isolated PCL injuries [8, 9, 12]. This variation is seen in both operative and nonoperative treatment strategies with distinct differences in rehabilitation protocols, graft selection and surgical technique. With the study cohort, variation was observed both within and across treatment groups. These differences were most notable with respect to initiation of full weightbearing and isolated hamstring strengthening between operative and non‐operative cohorts. Other rehabilitation milestones were similar and release to sport participation was achieved at a mean of 7 months after treatment. Successful return to sport participation was seen in 73% of patients at cross‐sectional follow‐up with no difference between treatment groups, a rate that is comparable to prior reports in adult cohorts [9].

Operative and nonoperative strategies for the management of isolated PCL injuries have been described in the literature. Ultimate treatment choice is influenced by injury severity, concomitant injury (e.g., meniscal injury), patient age and surgeon preference. Direct comparisons of these strategies are limited to the adult literature. However, a recent study by Rupp et al. compared operative vs. nonoperative management of paediatric PCL injuries (with and without concomitant meniscal injury) across 48 patients (49 knees) [7]. Patients with isolated PCL injury (*N* = 37) had similar results across all treatment cohorts (operative, non‐operative, and non‐operative to operative) [7]. However, patients with concomitant meniscal injury treated with initial non‐operative management and eventual operative management demonstrated significantly lower Lysholm scores than those with concomitant meniscus injury initially treated operatively [7]. The only complication reported in this study was related to patient noncompliance [7]. The average long‐term Lysholm scores were 92.5 ± 12.7 in the nonoperative cohort, and 85.3 ± 18.9 in the operative cohort (*p* = .072 between groups) [7]. Overall, the findings from the present study appear to concur with Rupp et al.'s results and support the management of paediatric isolated PCL injury with both operative and nonoperative strategies [7].

Scarcella et al. [8] recently summarised the outcomes of paediatric PCL reconstruction in a systematic review including 43 PCL injuries in 42 patients across four studies [4, 5, 14, 17]. Complications across all studies were uncommon with only one case of failed reconstruction requiring revision and three cases of arthrofibrosis, and one study identified leg length discrepancy in late follow‐up for a single patient [14]. wo studies reported Pedi‐IKDC scores with an aggregated mean of 80 at 56.3 months follow‐up while three studies reported Lysholm scores with an aggregated mean of 85.15 at a mean 51.5 months. These scores appear comparable to the scores observed in this study's operative and nonoperative cohorts at a similar timepoint. No studies among paediatric patients evaluated PROMIS score for direct comparison; however, the values observed in this study were within normative ranges.

Within the adult literature, Schroven et al. identified 5197 patients across 27 studies examining operative and nonoperative management of this injury. The mean age of patients was 29.5 years with the youngest patient included being 15 years old. While both treatment modalities yielded satisfactory outcome scores and successful return to sport in the majority of patients, operative treatment was associated with reduced laxity and osteoarthritis but also higher complication rates (3%–16%), including infection, graft failure, and arthrofibrosis [9]. These findings, when compared with the results of this study and the remainder of the paediatric literature, suggest that operative PCL treatment in adults may confer some benefits, but may also carry increased surgical risk.

Several limitations should be acknowledged in the interpretation of study findings. Importantly, the study cohort was small in size, compounded by a low rate of completion of the follow‐up survey, resulting in limited statistical power and generalisability. However, as there is a significant dearth of literature on the treatment of paediatric PCL injury, the study sample provides ample data to expand the present understanding of this injury and its treatment and potentially inform the study of prospective work on this topic. Furthermore, the study cohort had considerable variability in tear patterns and concomitant injuries, which limited more robust subgroup analysis. Additionally, treatment was based on surgeon preference due to limited available evidence guiding clinical‐decision making in the paediatric population for this injury. Thus, there was no standardisation of operative technique, postoperative restrictions, or rehabilitation protocols and this was evidenced by the findings observed in the study cohort. The retrospective nature of this study further limits consistency in outcome reporting including complications, rehabilitation progression, and sport clearance.Additionally, not all patients in the originally examined cohort completed the final cross‐sectional survey, but nonresponse analysis was not indicative of notable demographic biases in the participating cohort. Furthermore, there is a possibility of recall bias in cross‐sectional survey responses. However, patient‐reported outcome measures were related to the patient's current abilities, aiming to mitigate the amount of recall utilised in responses. Despite these acknowledged limitations, this study reports on one of the largest paediatric PCL cohorts published to date, provides comparative analyses among treatment modalities, and contains mid‐term cross‐sectional patient‐reported outcome data that bolsters the limited available evidence on this topic.

## CONCLUSION

Findings from this study provide valuable insight into the characteristics, treatment strategies, and outcomes of paediatric patients with isolated PCL injuries. Complications among operative and nonoperative treatment groups were uncommon, with no reports of PCL retear. Cross‐sectional assessment at a mean of nearly 5 years from injury in a limited but demographically‐similar subcohort of patients demonstrated normative values in patient‐reported and knee function outcomes scores in both groups that were comparable to prior paediatric PCL cohorts. While several limitations are acknowledged when interpreting these results, this study expands upon existing literature regarding paediatric PCL injuries and may help to inform evidence‐based guidelines for managing these injuries. The present study's results suggest that both operative and nonoperative strategies can both be reasonably considered for isolated PCL injuries in paediatric patients, and a shared‐decision making model with patients and families model may be preferred. Given that surgery is not without risk, when operative management is not otherwise dictated by contaminant injury or other factors, a nonoperative strategy appears to be a viable first‐line approach in this population. Future work should seek to evaluate both treatment strategies in a prospective manner to monitor for longer‐term outcomes and clarify factors that may impact the decision between treatment options.

## AUTHOR CONTRIBUTIONS

**Morgan Batley**: Substantial contributions to conception and design, data acquisition, data analysis, data interpretation, manuscript drafting, critical revision, and final approval of the version to be published. **Nathan Chaclas**: Substantial contributions to data acquisition, data analysis, data interpretation, manuscript drafting, critical revision, and final approval of the version to be published. **Katherine Ashe**: Substantial contributions to data interpretation, manuscript critical revision, and final approval of the version to be published. **Caroline L. Kim**: Substantial contributions to data interpretation, manuscript critical revisions, and final approval of the version to be published. **Theodore J. Ganley**: Substantial contributions to data interpretation, manuscript critical revision, and final approval of the version to be published. **Kathleen J. Maguire**: Substantial contributions to, data interpretation, manuscript critical revision, and final approval of the version to be published. **Brendan A. Williams**: Substantial contributions to conception and design, data acquisition, data analysis, data interpretation, manuscript drafting, critical revision, and final approval of the version to be published.

## CONFLICT OF INTEREST STATEMENT

The authors declare no conflicts of interest.

## ETHICS STATEMENT

All procedures performed in studies involving human participants were in accordance with the ethical standards of the institutional and/or national research committee and with the 1964 Helsinki Declaration and its later amendments or comparable ethical standards. The study was approved by the Institutional Review Board of the Children's Hospital of Philadelphia IRB# 22‐020555. Verbal consent was obtained for the cross‐sectional survey data collection. A waiver of consent was obtained for medical chart review.

## Supporting information

Supplementary Files.

## Data Availability

The data that supports the findings of this study are available on reasonable request from the corresponding author. The data are not publicly available due to privacy or ethical reasons.
